# *N*-[3-(Di­methyl­amino)­prop­yl]-*N*,*N*′,*N*′,*N*′′,*N*′′-penta­methyl­guanidinium tetra­phenyl­borate

**DOI:** 10.1107/S1600536813014906

**Published:** 2013-06-08

**Authors:** Ioannis Tiritiris

**Affiliations:** a Fakultät Chemie/Organische Chemie, Hochschule Aalen, Beethovenstrasse 1, D-73430 Aalen, Germany

## Abstract

In the title salt, C_11_H_27_N_4_^+^·C_24_H_20_B^−^, the C—N bond lengths in the central CN_3_ unit of the guanidinium ion are 1.333 (4), 1.334 (4) and 1.351 (4) Å, indicating partial double-bond character. The C atom of this unit is bonded to the three N atoms in a nearly ideal trigonal-planar geometry [N—C—N angles = 118.8 (3), 120.0 (3) and 121.2 (3)°] and the positive charge is delocalized in the CN_3_ plane. The bonds between the N atoms and the terminal C-methyl groups of the guanidinium moiety have values in the range 1.459 (4)–1.478 (4) Å, close to a typical single bond. In the crystal, there are C—H⋯π inter­actions between the guanidinium H atoms and the phenyl rings of the tetra­phenyl­borate ion. These inter­actions combine to form a ladder of linked chains of ions which runs parallel to the *c* axis.

## Related literature

For the synthesis of *N′′*-[3-(di­methyl­amino)­prop­yl]-*N*,*N*,*N′*,*N′*-tetra­methyl­guanidine, see: Tiritiris & Kantlehner (2012 ▶). For the crystal structures of alkali metal tetra­phenyl­borates, see: Behrens *et al.* (2012 ▶). For the crystal structure of *N*,*N*,*N′*,*N′*,*N′′*-tetra­methyl-*N′′*-[3-(tri­methyl­aza­nium­yl)prop­yl]guanid­in­ium bis­(tetra­phenyl­borate) acetone disolvate, see: Tiritiris (2013 ▶).
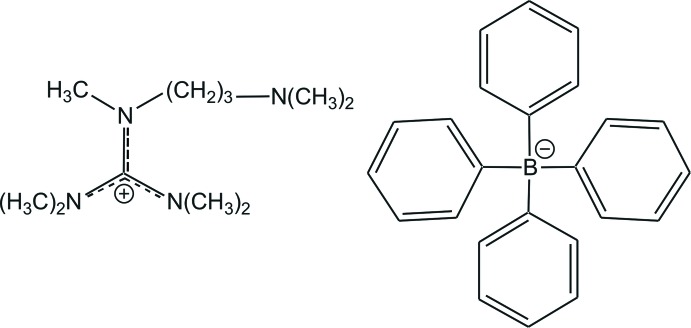


## Experimental

### 

#### Crystal data

C_11_H_27_N_4_^+^·C_24_H_20_B^−^
*M* *_r_* = 534.58
Orthorhombic, 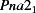

*a* = 20.5074 (7) Å
*b* = 15.4134 (5) Å
*c* = 9.8568 (3) Å
*V* = 3115.62 (17) Å^3^
*Z* = 4
Mo *K*α radiation
μ = 0.07 mm^−1^
*T* = 100 K
0.20 × 0.18 × 0.13 mm

#### Data collection

Bruker–Nonius KappaCCD diffractometer
7338 measured reflections
4035 independent reflections
3181 reflections with *I* > 2σ(*I*)
*R*_int_ = 0.049

#### Refinement

*R*[*F* ^2^ > 2σ(*F* ^2^)] = 0.057
*wR*(*F* ^2^) = 0.137
*S* = 1.05
4035 reflections
368 parameters
1 restraint
H-atom parameters constrained
Δρ_max_ = 0.48 e Å^−3^
Δρ_min_ = −0.20 e Å^−3^

### 

Data collection: *COLLECT* (Hooft, 2004 ▶); cell refinement: *SCALEPACK* (Otwinowski & Minor, 1997 ▶); data reduction: *SCALEPACK*; program(s) used to solve structure: *SHELXS97* (Sheldrick, 2008 ▶); program(s) used to refine structure: *SHELXL97* (Sheldrick, 2008 ▶); molecular graphics: *DIAMOND* (Brandenburg & Putz, 2005 ▶); software used to prepare material for publication: *SHELXL97*.

## Supplementary Material

Crystal structure: contains datablock(s) I, global. DOI: 10.1107/S1600536813014906/go2091sup1.cif

Structure factors: contains datablock(s) I. DOI: 10.1107/S1600536813014906/go2091Isup2.hkl

Additional supplementary materials:  crystallographic information; 3D view; checkCIF report

## Figures and Tables

**Table 1:** Hydrogen-bond geometry (Å, °) *Cg*1, *Cg*2 and *Cg*3 are the centroids of the C30–C35, C18–C23 and C24–C29 rings, respectively.

*D*—H⋯*A*	*D*—H	H⋯*A*	*D*⋯*A*	*D*—H⋯*A*
C2—H2*C*⋯*Cg*1^i^	0.98	2.48	3.425 (1)	162
C7—H7*A*⋯*Cg*2^ii^	0.99	2.84	3.821 (1)	170
C3—H3*A*⋯*Cg*2^i^	0.98	2.89	3.680 (1)	138
C9—H9*A*⋯*Cg*3^iii^	0.99	2.82	3.610 (1)	136

Symmetry codes: (i) 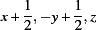; (ii) 
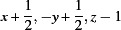
; (iii) 
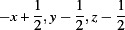
.

**Fig. 1.**
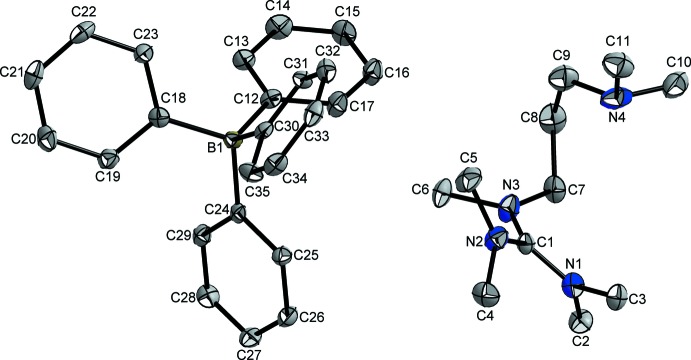
The structure of the title compound with displacement ellipsoids at the 50% probability level. All hydrogen atoms were omitted for the sake of clarity.

**Fig. 2.**
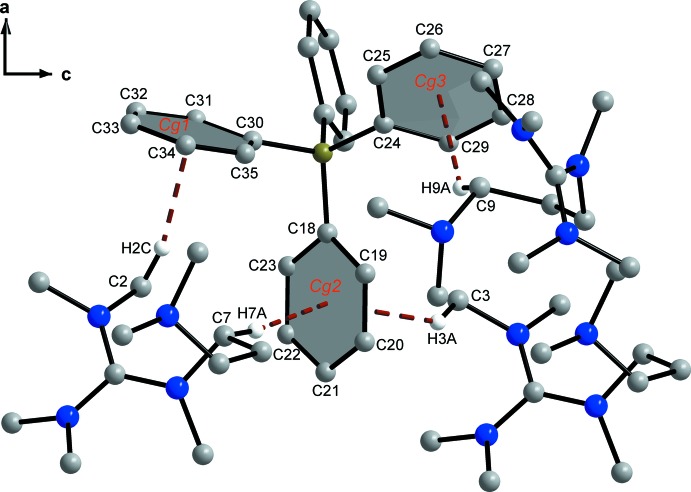
C–H···π interactions (brown dashed lines) between the hydrogen atoms of the guanidinium ion and the phenyl carbon atoms (centroids) of one tetraphenylborate ion.

## supplementary crystallographic information

### Comment

ω-Aminoalkylguanidines like *N''*-[3-(dimethylamino)propyl]- *N*,*N*,*N'*,*N'*-tetramethylguanidine (I) (Tiritiris & Kantlehner, 2012), in which two nitrogen atoms with different basicity are present, are considered as ambident nucleophiles. Electrophiles can attack at both, on the imine nitrogen of the guanidine function, as well as on the nitrogen atom of the (dimethylamino)propyl group. By alkylation of (I) with only one equivalent dimethyl sulfate, methylation occurs preferentially at the guanidine nitrogen atom, because it is the most basic site. The exclusion of moisture and the use of absolutely acid free dimethyl sulfate, is in this reaction very essential. Otherwise in first step protonation of the guanidine nitrogen atom occurs, followed by methylation of the (dimethylamino)propyl group, resulting in the dicationic *N*,*N*,*N'*,*N'*-tetramethyl-*N''*-[3- (trimethylazaniumyl)propyl]guanidinium ion (Tiritiris, 2013) as the main product. In fact, the reaction in wet solvents and the presence of acid traces, yields salt mixtures consisting of monocationic and dicationic species, which cannot be easily separated from each other. When performing the reaction under anhydrous conditions, the obtained waxy monomethylated methyl sulfate salt was converted after subsequent anion exchange with sodium tetraphenylborate to the crystalline title compound, whose X-ray structure is presented here.

According to the structure analysis, the C1–N1 bond of the the CN_3_ unit is 1.351 (4) Å, C1–N2 = 1.334 (4) Å and C1–N3 = 1.333 (4) Å, showing partial double-bond character. The N–C1–N angles are: 118.8 (3)° (N1–C1–N2), 120.0 (3)° (N1–C1–N3) and 121.2 (3)° (N2–C1–N3), which indicates a nearly ideal trigonal-planar surrounding of the carbon centre by the nitrogen atoms. The positive charge is completely delocalized on the CN_3_ plane (Fig. 1). The bonds between the N atoms and the terminal *C*-methyl groups of the guanidinium moiety all have values in the range 1.459 (4)–1.478 (4) Å, close to a typical single bond. The C–N bond lengths in the (dimethylamino)propyl group range from 1.437 (6) to 1.489 (6)Å. The bond lengths and angles in the tetraphenylborate ions are in good agreement with the data from the crystal structure analysis of the alkali metal tetraphenylborates (Behrens *et al.*, 2012). C–H···π interactions between the guanidinium hydrogen atoms of –N(CH_3_)_2_ and –CH_2_ groups and the phenyl carbon atoms (centroids) of the tetraphenylborate ion are present (Fig. 2), ranging from 2.48 to 2.89 Å (Tab. 1). These interactions combine to form a ladder of linked chains of ions which runs parallel to the *c* axis.

### Experimental

The title compound was obtained by reaction of *N*''-[3-(dimethylamino)propyl]-*N*,*N*,*N'*,*N'*-tetramethylguanidine (Tiritiris & Kantlehner, 2012) with one equivalent of freshly distilled dimethyl sulfate in anhydrous acetonitrile at room temperature. After evaporation of the solvent the crude *N*,*N*,*N'*,*N'*,*N''*-pentamethyl-*N''*-[3-(dimethylamino)propyl]-guanidinium methyl sulfate (II) was washed with diethylether and dried *in vacuo*. 1.0 g (2.8 mmol) of (II) was dissolved in 20 ml acetonitrile and 0.96 g (2.8 mmol) of sodium tetraphenylborate in 20 ml acetonitrile were added. After stirring for one hour at room temperature, the precipitated sodium methyl sulfate was filtered off. The title compound crystallized from a saturated acetone solution after several weeks at 273 K, forming colorless single crystals. Yield: 1.15 g (76.8%).

### Refinement

The title compound crystallizes in the non-centrosymmetric space group *Pna2_1_*; however, in the absence of significant anomalous scattering effects, the Flack parameter is essentially meaningless. Accordingly, Friedel pairs were merged. The hydrogen atoms of the methyl groups were allowed to rotate with a fixed angle around the C–N bond to best fit the experimental electron density, with *U*_iso_(H) set to 1.5 *U*_eq_(C) and d(C—H) = 0.98 Å. The remaining H atoms were placed in calculated positions with d(C—H) = 0.99 Å (H atoms in CH_2_ groups) and (C—H) = 0.95 Å (H atoms in aromatic rings). They were included in the refinement in the riding model approximation, with *U*_iso_(H) set to 1.2 *U*_eq_(C).

### Crystal data

**Table:**

C_11_H_27_N_4_^+^·C_24_H_20_B^−^	*F*(000) = 1160
*M**_r_* = 534.58	*D*_x_ = 1.140 Mg m^−^^3^
Orthorhombic, *P**n**a*2_1_	Mo *K*α radiation, λ = 0.71073 Å
Hall symbol: P 2c -2n	Cell parameters from 4121 reflections
*a* = 20.5074 (7) Å	θ = 0.4–28.3°
*b* = 15.4134 (5) Å	µ = 0.07 mm^−^^1^
*c* = 9.8568 (3) Å	*T* = 100 K
*V* = 3115.62 (17) Å^3^	Block, colorless
*Z* = 4	0.20 × 0.18 × 0.13 mm

### Data collection

**Table:**

Bruker–Nonius KappaCCD diffractometer	3181 reflections with *I* > 2σ(*I*)
Radiation source: sealed tube	*R*_int_ = 0.049
Graphite monochromator	θ_max_ = 28.3°, θ_min_ = 2.7°
φ scans, and ω scans	*h* = −27→27
7338 measured reflections	*k* = −20→20
4035 independent reflections	*l* = −13→12

### Refinement

**Table:**

Refinement on *F*^2^	Primary atom site location: structure-invariant direct methods
Least-squares matrix: full	Secondary atom site location: difference Fourier map
*R*[*F*^2^ > 2σ(*F*^2^)] = 0.057	Hydrogen site location: difference Fourier map
*wR*(*F*^2^) = 0.137	H-atom parameters constrained
*S* = 1.05	*w* = 1/[σ^2^(*F*_o_^2^) + (0.0694*P*)^2^ + 1.0225*P*] where *P* = (*F*_o_^2^ + 2*F*_c_^2^)/3
4035 reflections	(Δ/σ)_max_ < 0.001
368 parameters	Δρ_max_ = 0.48 e Å^−^^3^
1 restraint	Δρ_min_ = −0.20 e Å^−^^3^

### Special details

**Table:**

Geometry. All e.s.d.'s (except the e.s.d. in the dihedral angle between two l.s. planes) are estimated using the full covariance matrix. The cell e.s.d.'s are taken into account individually in the estimation of e.s.d.'s in distances, angles and torsion angles; correlations between e.s.d.'s in cell parameters are only used when they are defined by crystal symmetry. An approximate (isotropic) treatment of cell e.s.d.'s is used for estimating e.s.d.'s involving l.s. planes.
Refinement. Refinement of *F*^2^ against ALL reflections. The weighted *R*-factor *wR* and goodness of fit *S* are based on *F*^2^, conventional *R*-factors *R* are based on *F*, with *F* set to zero for negative *F*^2^. The threshold expression of *F*^2^ > σ(*F*^2^) is used only for calculating *R*-factors(gt) *etc*. and is not relevant to the choice of reflections for refinement. *R*-factors based on *F*^2^ are statistically about twice as large as those based on *F*, and *R*- factors based on ALL data will be even larger.

### Fractional atomic coordinates and isotropic or equivalent isotropic displacement parameters (Å^2^)

**Table:**

	*x*	*y*	*z*	*U*_iso_*/*U*_eq_	
C1	0.39908 (13)	0.30671 (18)	0.1123 (3)	0.0180 (6)	
N1	0.45724 (13)	0.34774 (16)	0.1064 (3)	0.0244 (6)	
N2	0.35484 (13)	0.32310 (18)	0.0164 (3)	0.0268 (6)	
N3	0.38671 (13)	0.25113 (19)	0.2126 (3)	0.0286 (6)	
C2	0.48919 (16)	0.3777 (2)	0.2309 (3)	0.0264 (7)	
H2A	0.4590	0.3721	0.3074	0.040*	
H2B	0.5019	0.4387	0.2205	0.040*	
H2C	0.5281	0.3425	0.2480	0.040*	
C3	0.48916 (15)	0.3656 (2)	−0.0229 (3)	0.0239 (7)	
H3A	0.4658	0.3357	−0.0960	0.036*	
H3B	0.5343	0.3449	−0.0196	0.036*	
H3C	0.4888	0.4282	−0.0400	0.036*	
C4	0.34857 (17)	0.4102 (2)	−0.0391 (4)	0.0289 (7)	
H4A	0.3756	0.4504	0.0136	0.043*	
H4B	0.3029	0.4286	−0.0344	0.043*	
H4C	0.3630	0.4104	−0.1339	0.043*	
C5	0.30700 (16)	0.2586 (2)	−0.0301 (4)	0.0322 (8)	
H5A	0.3196	0.2010	0.0029	0.048*	
H5B	0.3057	0.2583	−0.1295	0.048*	
H5C	0.2638	0.2736	0.0054	0.048*	
C6	0.32119 (17)	0.2457 (3)	0.2736 (4)	0.0354 (8)	
H6A	0.2932	0.2908	0.2345	0.053*	
H6B	0.3244	0.2542	0.3719	0.053*	
H6C	0.3023	0.1885	0.2548	0.053*	
C7	0.43937 (16)	0.2002 (2)	0.2769 (4)	0.0306 (7)	
H7A	0.4467	0.2219	0.3702	0.037*	
H7B	0.4803	0.2084	0.2250	0.037*	
C8	0.42283 (19)	0.1043 (2)	0.2822 (4)	0.0373 (9)	
H8A	0.3845	0.0964	0.3421	0.045*	
H8B	0.4599	0.0730	0.3239	0.045*	
C9	0.40794 (19)	0.0629 (2)	0.1454 (4)	0.0391 (9)	
H9A	0.4075	−0.0010	0.1563	0.047*	
H9B	0.3638	0.0810	0.1163	0.047*	
N4	0.45421 (17)	0.08554 (19)	0.0396 (3)	0.0390 (8)	
C10	0.5176 (2)	0.0427 (3)	0.0700 (5)	0.0482 (11)	
H10A	0.5113	−0.0203	0.0750	0.072*	
H10B	0.5344	0.0639	0.1569	0.072*	
H10C	0.5490	0.0561	−0.0021	0.072*	
C11	0.4322 (2)	0.0575 (3)	−0.0917 (4)	0.0491 (11)	
H11A	0.4265	−0.0057	−0.0913	0.074*	
H11B	0.4645	0.0735	−0.1604	0.074*	
H11C	0.3905	0.0854	−0.1128	0.074*	
B1	0.12881 (15)	0.2061 (2)	0.5818 (3)	0.0159 (6)	
C12	0.16933 (14)	0.12719 (18)	0.6527 (3)	0.0173 (6)	
C13	0.14514 (15)	0.07030 (18)	0.7512 (3)	0.0209 (6)	
H13	0.1010	0.0762	0.7787	0.025*	
C14	0.18295 (16)	0.0051 (2)	0.8113 (3)	0.0270 (7)	
H14	0.1642	−0.0323	0.8773	0.032*	
C15	0.24774 (17)	−0.0048 (2)	0.7742 (4)	0.0324 (8)	
H15	0.2736	−0.0492	0.8136	0.039*	
C16	0.27425 (17)	0.0512 (2)	0.6787 (4)	0.0328 (8)	
H16	0.3187	0.0453	0.6529	0.039*	
C17	0.23622 (14)	0.1154 (2)	0.6208 (3)	0.0246 (7)	
H17	0.2558	0.1534	0.5568	0.029*	
C18	0.04911 (14)	0.19818 (17)	0.6052 (3)	0.0177 (6)	
C19	0.00849 (14)	0.26899 (19)	0.6360 (3)	0.0220 (6)	
H19	0.0278	0.3242	0.6509	0.026*	
C20	−0.05945 (15)	0.2613 (2)	0.6456 (4)	0.0271 (7)	
H20	−0.0851	0.3108	0.6668	0.033*	
C21	−0.08949 (15)	0.1820 (2)	0.6244 (4)	0.0272 (7)	
H21	−0.1355	0.1766	0.6312	0.033*	
C22	−0.05143 (15)	0.1109 (2)	0.5932 (3)	0.0254 (7)	
H22	−0.0713	0.0560	0.5783	0.031*	
C23	0.01633 (14)	0.11928 (19)	0.5835 (3)	0.0212 (6)	
H23	0.0413	0.0694	0.5612	0.025*	
C24	0.15858 (13)	0.29536 (17)	0.6477 (3)	0.0158 (6)	
C25	0.20552 (14)	0.34705 (18)	0.5837 (3)	0.0213 (6)	
H25	0.2196	0.3312	0.4953	0.026*	
C26	0.23223 (14)	0.4200 (2)	0.6432 (3)	0.0242 (7)	
H26	0.2633	0.4535	0.5946	0.029*	
C27	0.21415 (14)	0.44452 (19)	0.7727 (3)	0.0233 (6)	
H27	0.2320	0.4951	0.8132	0.028*	
C28	0.16949 (14)	0.39393 (19)	0.8422 (3)	0.0211 (6)	
H28	0.1573	0.4090	0.9322	0.025*	
C29	0.14233 (14)	0.32109 (18)	0.7808 (3)	0.0192 (6)	
H29	0.1117	0.2874	0.8304	0.023*	
C30	0.13792 (14)	0.20459 (18)	0.4149 (3)	0.0167 (6)	
C31	0.15854 (14)	0.13116 (19)	0.3429 (3)	0.0186 (6)	
H31	0.1696	0.0804	0.3925	0.022*	
C32	0.16356 (15)	0.1292 (2)	0.2026 (3)	0.0237 (7)	
H32	0.1767	0.0772	0.1586	0.028*	
C33	0.14963 (14)	0.2019 (2)	0.1260 (3)	0.0241 (7)	
H33	0.1541	0.2013	0.0301	0.029*	
C34	0.12899 (16)	0.2758 (2)	0.1937 (3)	0.0274 (7)	
H34	0.1188	0.3265	0.1431	0.033*	
C35	0.12292 (15)	0.27719 (19)	0.3343 (3)	0.0234 (7)	
H35	0.1082	0.3288	0.3772	0.028*	

### Atomic displacement parameters (Å^2^)

**Table:**

	*U*^11^	*U*^22^	*U*^33^	*U*^12^	*U*^13^	*U*^23^
C1	0.0173 (13)	0.0200 (13)	0.0165 (14)	0.0010 (11)	0.0008 (11)	0.0009 (12)
N1	0.0237 (13)	0.0283 (13)	0.0213 (13)	0.0020 (11)	−0.0011 (11)	0.0011 (12)
N2	0.0239 (14)	0.0282 (13)	0.0283 (15)	−0.0020 (11)	−0.0049 (12)	0.0045 (12)
N3	0.0209 (13)	0.0362 (15)	0.0288 (14)	0.0002 (12)	−0.0016 (12)	0.0066 (13)
C2	0.0236 (16)	0.0304 (17)	0.0252 (17)	0.0002 (14)	−0.0095 (13)	−0.0042 (14)
C3	0.0219 (15)	0.0306 (16)	0.0194 (15)	0.0012 (13)	0.0012 (12)	0.0044 (13)
C4	0.0316 (17)	0.0265 (16)	0.0286 (17)	0.0045 (14)	−0.0103 (14)	0.0040 (14)
C5	0.0233 (16)	0.0365 (18)	0.0370 (19)	−0.0069 (15)	−0.0103 (15)	−0.0019 (16)
C6	0.0273 (17)	0.051 (2)	0.0280 (17)	−0.0036 (16)	0.0070 (15)	0.0051 (18)
C7	0.0288 (17)	0.0380 (18)	0.0250 (16)	0.0079 (14)	−0.0015 (14)	0.0122 (16)
C8	0.0383 (19)	0.0395 (19)	0.034 (2)	0.0062 (16)	0.0064 (17)	0.0152 (17)
C9	0.042 (2)	0.0292 (17)	0.046 (2)	−0.0023 (16)	0.0041 (19)	0.0113 (18)
N4	0.054 (2)	0.0276 (15)	0.0353 (17)	−0.0149 (15)	0.0125 (15)	0.0025 (13)
C10	0.042 (2)	0.054 (2)	0.049 (3)	−0.015 (2)	0.0106 (19)	−0.006 (2)
C11	0.062 (3)	0.039 (2)	0.045 (3)	0.001 (2)	0.002 (2)	0.003 (2)
B1	0.0161 (14)	0.0141 (14)	0.0176 (16)	−0.0001 (12)	−0.0008 (12)	0.0019 (13)
C12	0.0186 (14)	0.0176 (13)	0.0158 (14)	−0.0030 (11)	−0.0029 (11)	0.0021 (11)
C13	0.0238 (15)	0.0208 (14)	0.0182 (16)	0.0007 (12)	0.0009 (12)	0.0040 (12)
C14	0.0327 (17)	0.0276 (15)	0.0206 (16)	−0.0004 (14)	−0.0018 (13)	0.0096 (13)
C15	0.0340 (18)	0.0330 (17)	0.0302 (17)	0.0073 (15)	−0.0065 (15)	0.0133 (16)
C16	0.0231 (16)	0.0389 (19)	0.036 (2)	0.0029 (15)	−0.0030 (14)	0.0091 (16)
C17	0.0180 (14)	0.0280 (15)	0.0277 (17)	0.0012 (12)	−0.0050 (13)	0.0097 (14)
C18	0.0195 (13)	0.0180 (13)	0.0156 (14)	0.0008 (11)	−0.0025 (12)	0.0024 (11)
C19	0.0184 (14)	0.0198 (13)	0.0278 (16)	−0.0014 (11)	−0.0039 (13)	0.0023 (13)
C20	0.0187 (15)	0.0292 (16)	0.0335 (18)	0.0067 (13)	−0.0038 (14)	0.0030 (15)
C21	0.0155 (13)	0.0408 (17)	0.0254 (17)	−0.0022 (13)	−0.0013 (13)	0.0053 (15)
C22	0.0241 (15)	0.0283 (15)	0.0239 (16)	−0.0128 (13)	−0.0002 (13)	0.0014 (14)
C23	0.0196 (14)	0.0205 (14)	0.0236 (16)	−0.0039 (12)	0.0026 (12)	−0.0019 (12)
C24	0.0116 (12)	0.0169 (12)	0.0188 (14)	0.0024 (10)	−0.0028 (11)	0.0037 (11)
C25	0.0185 (14)	0.0218 (14)	0.0235 (16)	−0.0036 (12)	0.0024 (12)	−0.0070 (13)
C26	0.0182 (14)	0.0263 (15)	0.0282 (17)	−0.0060 (12)	0.0015 (13)	−0.0022 (14)
C27	0.0192 (14)	0.0234 (14)	0.0273 (16)	−0.0023 (12)	−0.0078 (14)	−0.0056 (14)
C28	0.0226 (14)	0.0260 (15)	0.0148 (14)	0.0019 (12)	−0.0040 (12)	−0.0027 (12)
C29	0.0162 (13)	0.0213 (13)	0.0202 (15)	0.0004 (11)	−0.0023 (12)	0.0031 (13)
C30	0.0140 (13)	0.0167 (13)	0.0193 (14)	−0.0050 (11)	−0.0014 (11)	0.0029 (12)
C31	0.0187 (14)	0.0148 (13)	0.0223 (15)	0.0010 (11)	−0.0001 (12)	0.0021 (12)
C32	0.0193 (15)	0.0291 (16)	0.0228 (16)	0.0027 (13)	0.0013 (12)	−0.0035 (14)
C33	0.0171 (13)	0.0365 (17)	0.0188 (15)	−0.0040 (12)	−0.0024 (12)	0.0027 (14)
C34	0.0269 (17)	0.0299 (17)	0.0254 (17)	−0.0030 (14)	−0.0051 (13)	0.0138 (14)
C35	0.0286 (16)	0.0162 (14)	0.0253 (17)	0.0045 (13)	−0.0024 (13)	0.0040 (13)

### Geometric parameters (Å, º)

**Table:**

C1—N3	1.333 (4)	C12—C13	1.400 (4)
C1—N2	1.334 (4)	C12—C17	1.419 (4)
C1—N1	1.351 (4)	C13—C14	1.401 (4)
N1—C3	1.459 (4)	C13—H13	0.9500
N1—C2	1.466 (4)	C14—C15	1.386 (5)
N2—C4	1.456 (4)	C14—H14	0.9500
N2—C5	1.470 (4)	C15—C16	1.387 (5)
N3—C6	1.474 (4)	C15—H15	0.9500
N3—C7	1.478 (4)	C16—C17	1.384 (4)
C2—H2A	0.9800	C16—H16	0.9500
C2—H2B	0.9800	C17—H17	0.9500
C2—H2C	0.9800	C18—C23	1.406 (4)
C3—H3A	0.9800	C18—C19	1.406 (4)
C3—H3B	0.9800	C19—C20	1.402 (4)
C3—H3C	0.9800	C19—H19	0.9500
C4—H4A	0.9800	C20—C21	1.385 (5)
C4—H4B	0.9800	C20—H20	0.9500
C4—H4C	0.9800	C21—C22	1.380 (5)
C5—H5A	0.9800	C21—H21	0.9500
C5—H5B	0.9800	C22—C23	1.399 (4)
C5—H5C	0.9800	C22—H22	0.9500
C6—H6A	0.9800	C23—H23	0.9500
C6—H6B	0.9800	C24—C25	1.400 (4)
C6—H6C	0.9800	C24—C29	1.410 (4)
C7—C8	1.518 (5)	C25—C26	1.381 (4)
C7—H7A	0.9900	C25—H25	0.9500
C7—H7B	0.9900	C26—C27	1.382 (5)
C8—C9	1.523 (6)	C26—H26	0.9500
C8—H8A	0.9900	C27—C28	1.384 (4)
C8—H8B	0.9900	C27—H27	0.9500
C9—N4	1.453 (5)	C28—C29	1.392 (4)
C9—H9A	0.9900	C28—H28	0.9500
C9—H9B	0.9900	C29—H29	0.9500
N4—C11	1.437 (6)	C30—C31	1.401 (4)
N4—C10	1.489 (6)	C30—C35	1.407 (4)
C10—H10A	0.9800	C31—C32	1.387 (4)
C10—H10B	0.9800	C31—H31	0.9500
C10—H10C	0.9800	C32—C33	1.382 (5)
C11—H11A	0.9800	C32—H32	0.9500
C11—H11B	0.9800	C33—C34	1.386 (5)
C11—H11C	0.9800	C33—H33	0.9500
B1—C12	1.630 (4)	C34—C35	1.392 (5)
B1—C24	1.640 (4)	C34—H34	0.9500
B1—C18	1.655 (4)	C35—H35	0.9500
B1—C30	1.656 (4)		
			
N3—C1—N2	121.2 (3)	C24—B1—C18	112.0 (2)
N3—C1—N1	120.0 (3)	C12—B1—C30	111.0 (2)
N2—C1—N1	118.8 (3)	C24—B1—C30	111.3 (2)
C1—N1—C3	121.5 (3)	C18—B1—C30	104.4 (2)
C1—N1—C2	120.4 (3)	C13—C12—C17	114.6 (3)
C3—N1—C2	118.1 (2)	C13—C12—B1	125.7 (3)
C1—N2—C4	120.1 (3)	C17—C12—B1	119.6 (2)
C1—N2—C5	123.1 (3)	C12—C13—C14	123.2 (3)
C4—N2—C5	116.6 (3)	C12—C13—H13	118.4
C1—N3—C6	120.8 (3)	C14—C13—H13	118.4
C1—N3—C7	121.4 (3)	C15—C14—C13	119.8 (3)
C6—N3—C7	117.5 (3)	C15—C14—H14	120.1
N1—C2—H2A	109.5	C13—C14—H14	120.1
N1—C2—H2B	109.5	C14—C15—C16	119.1 (3)
H2A—C2—H2B	109.5	C14—C15—H15	120.5
N1—C2—H2C	109.5	C16—C15—H15	120.5
H2A—C2—H2C	109.5	C17—C16—C15	120.3 (3)
H2B—C2—H2C	109.5	C17—C16—H16	119.9
N1—C3—H3A	109.5	C15—C16—H16	119.9
N1—C3—H3B	109.5	C16—C17—C12	123.0 (3)
H3A—C3—H3B	109.5	C16—C17—H17	118.5
N1—C3—H3C	109.5	C12—C17—H17	118.5
H3A—C3—H3C	109.5	C23—C18—C19	114.9 (3)
H3B—C3—H3C	109.5	C23—C18—B1	121.0 (2)
N2—C4—H4A	109.5	C19—C18—B1	123.9 (2)
N2—C4—H4B	109.5	C20—C19—C18	122.5 (3)
H4A—C4—H4B	109.5	C20—C19—H19	118.7
N2—C4—H4C	109.5	C18—C19—H19	118.7
H4A—C4—H4C	109.5	C21—C20—C19	120.5 (3)
H4B—C4—H4C	109.5	C21—C20—H20	119.8
N2—C5—H5A	109.5	C19—C20—H20	119.8
N2—C5—H5B	109.5	C22—C21—C20	118.9 (3)
H5A—C5—H5B	109.5	C22—C21—H21	120.6
N2—C5—H5C	109.5	C20—C21—H21	120.6
H5A—C5—H5C	109.5	C21—C22—C23	120.2 (3)
H5B—C5—H5C	109.5	C21—C22—H22	119.9
N3—C6—H6A	109.5	C23—C22—H22	119.9
N3—C6—H6B	109.5	C22—C23—C18	123.0 (3)
H6A—C6—H6B	109.5	C22—C23—H23	118.5
N3—C6—H6C	109.5	C18—C23—H23	118.5
H6A—C6—H6C	109.5	C25—C24—C29	115.0 (3)
H6B—C6—H6C	109.5	C25—C24—B1	123.7 (3)
N3—C7—C8	111.6 (3)	C29—C24—B1	121.1 (2)
N3—C7—H7A	109.3	C26—C25—C24	123.0 (3)
C8—C7—H7A	109.3	C26—C25—H25	118.5
N3—C7—H7B	109.3	C24—C25—H25	118.5
C8—C7—H7B	109.3	C25—C26—C27	120.6 (3)
H7A—C7—H7B	108.0	C25—C26—H26	119.7
C7—C8—C9	115.0 (3)	C27—C26—H26	119.7
C7—C8—H8A	108.5	C26—C27—C28	118.7 (3)
C9—C8—H8A	108.5	C26—C27—H27	120.6
C7—C8—H8B	108.5	C28—C27—H27	120.6
C9—C8—H8B	108.5	C27—C28—C29	120.3 (3)
H8A—C8—H8B	107.5	C27—C28—H28	119.9
N4—C9—C8	113.8 (3)	C29—C28—H28	119.9
N4—C9—H9A	108.8	C28—C29—C24	122.5 (3)
C8—C9—H9A	108.8	C28—C29—H29	118.8
N4—C9—H9B	108.8	C24—C29—H29	118.8
C8—C9—H9B	108.8	C31—C30—C35	115.0 (3)
H9A—C9—H9B	107.7	C31—C30—B1	123.3 (3)
C11—N4—C9	111.6 (3)	C35—C30—B1	121.7 (3)
C11—N4—C10	108.8 (3)	C32—C31—C30	123.0 (3)
C9—N4—C10	108.6 (3)	C32—C31—H31	118.5
N4—C10—H10A	109.5	C30—C31—H31	118.5
N4—C10—H10B	109.5	C33—C32—C31	120.7 (3)
H10A—C10—H10B	109.5	C33—C32—H32	119.6
N4—C10—H10C	109.5	C31—C32—H32	119.6
H10A—C10—H10C	109.5	C32—C33—C34	117.9 (3)
H10B—C10—H10C	109.5	C32—C33—H33	121.1
N4—C11—H11A	109.5	C34—C33—H33	121.1
N4—C11—H11B	109.5	C33—C34—C35	121.2 (3)
H11A—C11—H11B	109.5	C33—C34—H34	119.4
N4—C11—H11C	109.5	C35—C34—H34	119.4
H11A—C11—H11C	109.5	C34—C35—C30	122.1 (3)
H11B—C11—H11C	109.5	C34—C35—H35	119.0
C12—B1—C24	105.4 (2)	C30—C35—H35	119.0
C12—B1—C18	112.9 (2)		
			
N3—C1—N1—C3	143.5 (3)	C30—B1—C18—C19	−101.0 (3)
N2—C1—N1—C3	−36.6 (4)	C23—C18—C19—C20	0.6 (5)
N3—C1—N1—C2	−38.2 (4)	B1—C18—C19—C20	175.3 (3)
N2—C1—N1—C2	141.7 (3)	C18—C19—C20—C21	−0.1 (5)
N3—C1—N2—C4	143.5 (3)	C19—C20—C21—C22	−0.2 (5)
N1—C1—N2—C4	−36.4 (4)	C20—C21—C22—C23	0.0 (5)
N3—C1—N2—C5	−31.1 (5)	C21—C22—C23—C18	0.5 (5)
N1—C1—N2—C5	149.0 (3)	C19—C18—C23—C22	−0.8 (5)
N2—C1—N3—C6	−37.1 (5)	B1—C18—C23—C22	−175.6 (3)
N1—C1—N3—C6	142.8 (3)	C12—B1—C24—C25	98.6 (3)
N2—C1—N3—C7	149.9 (3)	C18—B1—C24—C25	−138.3 (3)
N1—C1—N3—C7	−30.2 (4)	C30—B1—C24—C25	−21.8 (4)
C1—N3—C7—C8	−129.3 (3)	C12—B1—C24—C29	−75.6 (3)
C6—N3—C7—C8	57.5 (4)	C18—B1—C24—C29	47.5 (3)
N3—C7—C8—C9	56.9 (4)	C30—B1—C24—C29	164.0 (2)
C7—C8—C9—N4	46.0 (4)	C29—C24—C25—C26	−2.6 (4)
C8—C9—N4—C11	−170.0 (3)	B1—C24—C25—C26	−177.0 (3)
C8—C9—N4—C10	70.0 (4)	C24—C25—C26—C27	1.3 (5)
C24—B1—C12—C13	106.9 (3)	C25—C26—C27—C28	0.9 (5)
C18—B1—C12—C13	−15.7 (4)	C26—C27—C28—C29	−1.6 (4)
C30—B1—C12—C13	−132.5 (3)	C27—C28—C29—C24	0.2 (4)
C24—B1—C12—C17	−69.3 (3)	C25—C24—C29—C28	1.8 (4)
C18—B1—C12—C17	168.2 (3)	B1—C24—C29—C28	176.5 (3)
C30—B1—C12—C17	51.4 (4)	C12—B1—C30—C31	19.6 (4)
C17—C12—C13—C14	−1.8 (4)	C24—B1—C30—C31	136.7 (3)
B1—C12—C13—C14	−178.2 (3)	C18—B1—C30—C31	−102.3 (3)
C12—C13—C14—C15	0.5 (5)	C12—B1—C30—C35	−162.8 (3)
C13—C14—C15—C16	0.7 (5)	C24—B1—C30—C35	−45.7 (4)
C14—C15—C16—C17	−0.5 (5)	C18—B1—C30—C35	75.3 (3)
C15—C16—C17—C12	−1.0 (5)	C35—C30—C31—C32	−0.4 (4)
C13—C12—C17—C16	2.1 (5)	B1—C30—C31—C32	177.3 (3)
B1—C12—C17—C16	178.7 (3)	C30—C31—C32—C33	1.6 (5)
C12—B1—C18—C23	−47.2 (4)	C31—C32—C33—C34	−1.6 (5)
C24—B1—C18—C23	−166.0 (3)	C32—C33—C34—C35	0.5 (5)
C30—B1—C18—C23	73.4 (3)	C33—C34—C35—C30	0.7 (5)
C12—B1—C18—C19	138.4 (3)	C31—C30—C35—C34	−0.7 (4)
C24—B1—C18—C19	19.6 (4)	B1—C30—C35—C34	−178.5 (3)

### Hydrogen-bond geometry (Å, º)

Cg1, Cg2 and Cg3 are the centroids of the C30–C35, C18–C23 and C24–C29 rings, respectively.

**Table:**

*D*—H···*A*	*D*—H	H···*A*	*D*···*A*	*D*—H···*A*
C2—H2*C*···*Cg*1^i^	0.98	2.48	3.425 (1)	162
C7—H7*A*···*Cg*2^ii^	0.99	2.84	3.821 (1)	170
C3—H3*A*···*Cg*2^i^	0.98	2.89	3.680 (1)	138
C9—H9*A*···*Cg*3^iii^	0.99	2.82	3.610 (1)	136

Symmetry codes: (i) *x*+1/2, −*y*+1/2, *z*; (ii) *x*+1/2, −*y*+1/2, *z*−1; (iii) −*x*+1/2, *y*−1/2, *z*−1/2.
